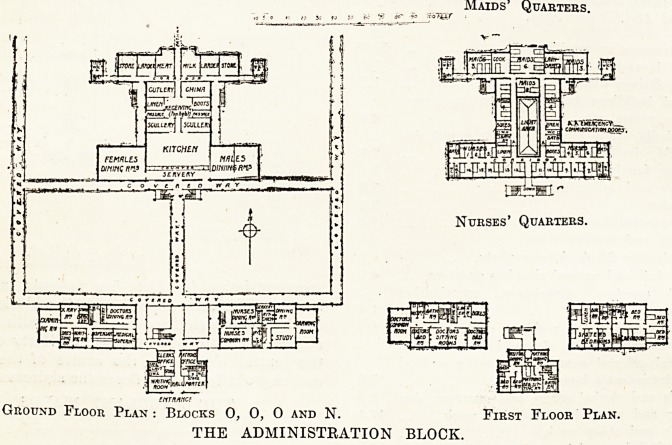# Sanatoria Buildings at £88 per Bed

**Published:** 1914-04-18

**Authors:** A. Alban H. Scott


### HOSPITAL ARCHITECTURE AND CONSTRUCTION.

### Sanatoria Buildings at £88 per bed.

By A. ALBAN H. SCOTT.

I HAVE read with very much interest the particulars which you have recently given in THE HOSPITAL with regard to sanatoria buildings. I have lately completed plans of a sanatorium for 100 beds, and after allowing what I think would be ample provision in the way of accommodation, I find that upon very close analysis of the cost properly designed buildings can be erected for £88 per bed. This is on the assumption that the ordinary patients are accommodated in pavilions on the lines of an ordinary hospital ward. If the hospital ward plan were not adopted, but rooms with corridor at the rear giving access to the rooms, the extra cost involved would be about £40 per bed, making the total cost about £128 per bed, and in this case item No. 1 in the following analysis should read £53.75 per cent.

| | | £ |
|---|---|---|
| 1. | Cost of wards or pavilions ... ... ... ... | 13.75 |
| 2. | Cost of lavatories connected therewith ... | 1.8 |
| 3. | Cost of fittings ditto ... ... ... ... | 1.2 |
| 4. | Attached buildings to ward, such as stores, drying rooms, etc. ... ... ... ... | 4.4 |
| 5. | Dining halls, writing rooms, which would also probably be used as recreation room ... | 2.8 |
| 6. | Buildings for laundry, generating plant, etc. | 3.7 |
| 7. | Mortuary and laboratories ... ... ... | 1.3 |
| 8. | Gate lodge ... ... ... ... ... ... | 2.5 |
| 9. | Accommodation for male staff ... ... ... | 1.4 |
| 10. | Corridors and covered ways ... ... ... | 5.0 |
| 11. | Administration block, kitchens, etc. ... ... | 4.6 |
| 12. | Female staff and nurses' sleeping quarters ... | 4.6 |
| 13. | Doctors' quarters, dispensary, examination rooms, etc. ... ... ... ... ... | 1.4 |
| 14. | Sleeping quarters for resident doctors ... | 1.4 |
| 15. | Nurses' common rooms, sisters' bedrooms, etc., medical officers' quarters, including medical superintendent's house ... ... ... ... | 3.8 |
| 16. | Matron's and clerks' offices and quarters, etc. | 1.6 |
| 17. | Heating and general engineering work ... | 17.1 |
| 18. | Drainage and other work ... ... ... | 5.0 |
| 19. | Electric lighting, wiring (plant included elsewhere) ... ... ... ... ... ... | 2.0 |
| 20. | General sundry items to buildings ... ... | 5.0 |
| 21. | Allow for contingent work such as paths, etc. | 3.0 |
| | Total ... ... ... | £87.35 |

Say, £88 per bed.

This estimate has been based on the plans here given, which may be of interest.

### THE PLANS IN DETAIL.

They are not put forward as ideal or the most economical proposals for sanatoria, as they have been prepared for a competition, and the formation of the plans and accommodation had to follow the very definite lines laid down in the particulars published. In this case very little latitude was given to architects. With regard to the block plan, this is perhaps a little cramped, but as the site is divided by a road, and as all the buildings, with the exception of the administration block, were, under the conditions, bound to be on one field, it was better to put the administration block on the same site. Personally, I think that the buildings should have occupied a site about twice the size shown on the plan.

### ROOMS AND AMBULANT BLOCK.

The size of the rooms in the ambulant block is as required by the conditions—namely, 9 feet by 9 feet by 9 feet high. The ambulant block consists of ground and first floors, with total

SANATORIUM·

BLOCK PLAN·

accommodation for forty-five patients in separate rooms. The centre portion of the block contains ten beds on each floor facing due south, and two side wings set off at an angle of thirty degrees to the central block. In the centre of the building on the ground and first floors the staircase is placed

THE AMBULANT WARDS.

BLOCKS C.C

at the right-hand side of the entrance, and the duty room on the left; and at the rear, facing north, a block of buildings immediately behind the centre, containing bathrooms, lavatories, etc., also lavatorium, boot, linen, and drying rooms, and housemaids' closet, together with separate lavatory accommodation for nurses.

The whole of the front elevations facing south have been so arranged that not less than 90 per cent. of the total area of the south frontage can be opened or closed at will.

### Constructional Details.

The construction is as follows:—

*Floors.*—The ground to be excavated to the necessary depths and made up to the required levels. Not less than 6 inches Portland cement concrete to be placed under all buildings. These to be prepared for and paved with jointless paving as used in many institutions, formed of asbestos, etc., finished in either grey, buff, green, or red colour.

*Walls.*—The walls to be rough-cast or other buildings purely in the form of barns without internal plastering; and, further, it would appear from the evidence published that heating in the pavilions or wards is absolutely necessary, and the above estimate allows for this, together with all the necessary baths.

Personally, I am quite confident that the total cost of construction of the sanatorium need not exceed the figures mentioned in the Departmental Committee's Report. Assuming that the buildings as above on the basis of 100 patients

| | £ |
|---|---|
| Cost £88 per head = ... ... ... ... ... | 8,800 |
| 50 acres of land at £80 per acre ... = £40 per bed. | 4,000 |
| Furniture at ... ... ... ... = £22 per bed. | 2,200 |
| Total for 100 beds ... ... ... | £15,000 |
| = £150 per bed, | |

Ground Floor Plan: Blocks O, O, O and N.

Maids' Quarters.

Nurses' Quarters.

First Floor Plan.

THE ADMINISTRATION BLOCK.

cement coat finish on the outside, and would be constructed of panels built between the reinforced concrete structural work in the form of columns and beams, each panel being formed of 3-inch brickwork on the outside with a cavity of 2 inches, and a panel on the inside filled in with concrete walls 2 inches thick, the inside walling plastered with two coats, the latter being finished smooth ready to receive decoration either in the form of distemper or, when the plastering is quite dry, paint and enamel.

*Roofs.*—These to be formed of reinforced concrete with special asphalte covering on the outside, the inside to be plastered as described for the walling.

### The Heating Question.

I feel sure that to obtain a sanatorium at the prices mentioned above, it is *not* necessary to have which comes within the cost mentioned in the Report of the Departmental Committee. I venture to suggest that a sanatorium built on the lines indicated above would be worthy of the name.

### The Wall Problem.

A hospital recently constructed on similar lines to the above has been erected within fifteen miles of Charing Cross, and has proved exceedingly satisfactory.

There is one other point which I think ought to be mentioned—namely, that it would undoubtedly be a great mistake to form the walls of any sanatorium building only 3 inches total thickness. Condensation would render the premises exceedingly unsatisfactory, and this trouble would certainly be increased if any of the buildings so constructed were in any way heated.

## Figures and Tables

**Figure f1:**
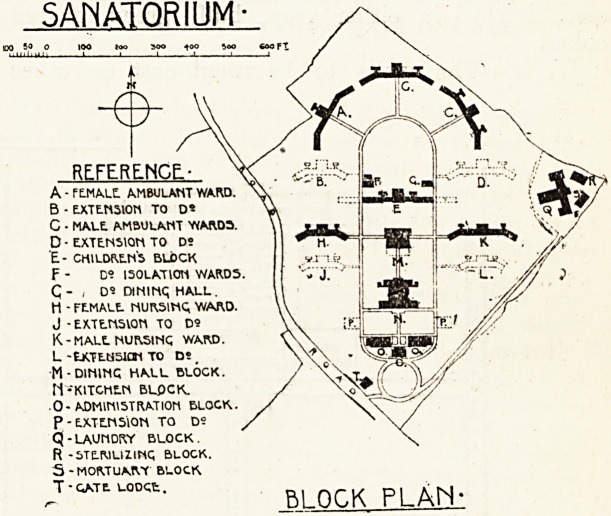


**Figure f2:**
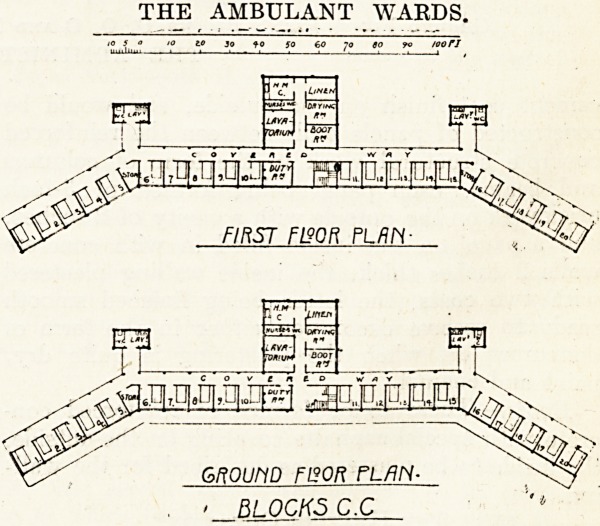


**Figure f3:**